# Supplementary material for: Gut Microbiome–Estrobolome Profile in Reproductive-Age Women with Endometriosis
Source: Int J Mol Sci. 2023 Nov 14;24(22):16301. doi: 10.3390/ijms242216301 (PMC10671785; doi:10.3390/ijms242216301)
Supplement: Supplementary file 1 [file ijms-24-16301-s001.zip › ijms-2662786-supplementary.pdf]

Supplemental Fig 1A

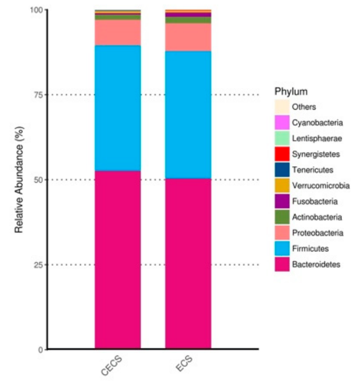

Supplemental Fig 1B

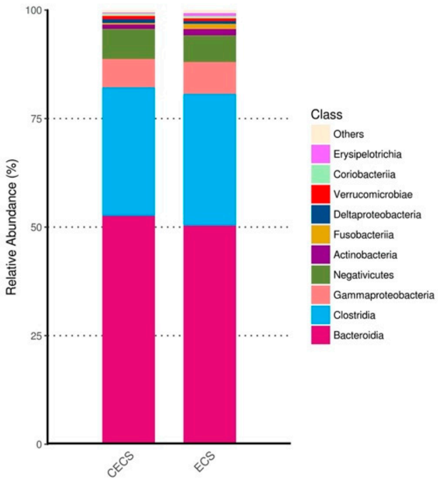

Supplemental Fig 1C

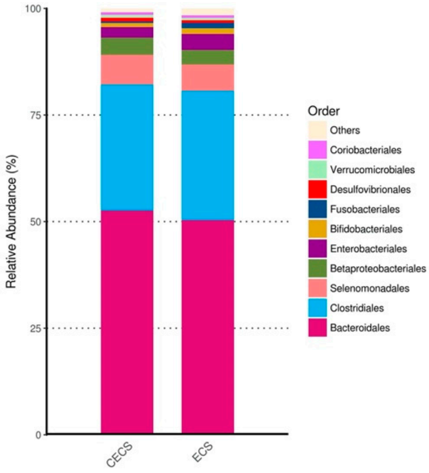

Supplemental Fig 1D

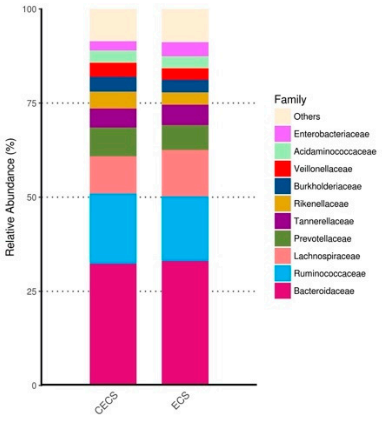

Supplemental Fig 1E

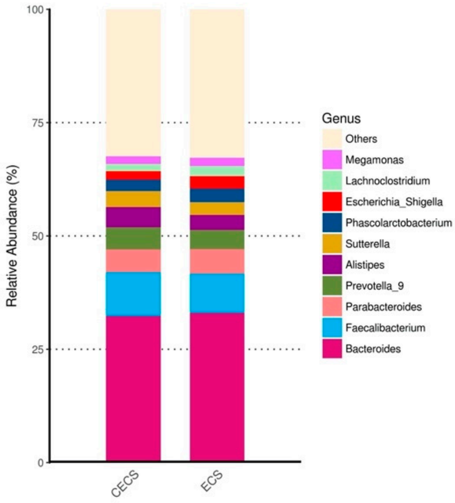

Supplemental Figure 1. Bar charts show the top 10 species with the highest relative abundance in each taxonomic level, with the bottom signifying the most abundant. (**A**) Phylum (**B**) Class (**C**) Order (**D**) Family (**E**) Genus. CECS = control, ECS = endometriosis.
